# Case Study of a MEMS Snap-Through Actuator: Modeling and Fabrication Considerations

**DOI:** 10.3390/mi13050654

**Published:** 2022-04-20

**Authors:** Zhichao Shi, Emile Martincic, Johan Moulin, Elie Lefeuvre, Frédéric Lamarque

**Affiliations:** 1 Sino-French Engineer School, Nanjing University of Science and Technology, 200 Xiaolingwei Street, Nanjing 210094, China; zhichao.shi@njust.edu.cn; 2 Centre de Nanosciences et de Nanotechnologies, CNRS, Université Paris-Saclay, 91120 Palaiseau, France; johan.moulin@c2n.upsaclay.fr (J.M.); elie.lefeuvre@c2n.upsaclay.fr (E.L.); 3 Roberval, CNRS, FRE 2012, Centre de Recherche Royallieu, Université de Technologie de Compiègne, Sorbonne Universités, 60203 Compiegne, France; frederic.lamarque@utc.fr

**Keywords:** MEMS, buckled beam micro-actuator, switching force, displacement characterization

## Abstract

MEMS actuators rely on the deformation of silicon structures. Using dimensions smaller than dozens of micrometers reveals that the micro-electro-mechanical systems (MEMS) actuators are affected by fabrication inaccuracies, leading to hardly predictable forces and/or actuation results. In this paper, MEMS bistable buckled beam actuators are presented. A series of structures based on pre-shaped buckled beams of lengths ranging from 2 to 4 mm, constant width of 5 μm and actuation stroke ranging from 20 to 100 μm was fabricated. Experimental data show a significant difference with predictions from a conventional analytical model. The model commonly used for buckled beams design assumes a rectangular beam section, but it is not the case of the fabricated beams. Furthermore, only symmetric buckling modes (mode 1, mode 3…) are supposed to exist during snap-through. In this paper, new analytical models have been developed on the basis of the models of the literature to consider the effective beam shape. The first improved analytical model enabled prediction of the MEMS buckled beams mechanical behavior in a 30% margin on the whole range of operation. A second model has been introduced to consider both the effective shape of the beam and centro-symmetric buckling modes. This refined model exhibits the partial suppression of buckling mode 2 by a central shuttle. Therefore, mode 2 and mode 3 coexist at the beginning and the end of snap-through, while mode 3 quickly vanishes due to increasing rotation of the central shuttle to leave exclusive presence of mode 2 near the mid-stroke. With this refined model, the effective force-displacement curve can be predicted in a margin reduced to a few percentages in the center zone of the response curve, allowing the accurate prediction of the position switch force. In addition, the proposed model allows accurate results to be reached with very small calculation time.

## 1. Introduction

Bistable micro-actuators are able to output a force and/or displacement on demand. They are used for switching applications when continuous actuation is not required. Since energy consumption and power supply are only needed during switches, bistable mechanical actuators fulfill these actuation requirements. They are used in a broad scope of micro-electro-mechanical systems (MEMS) applications, such as switches [1], clips [2], memory units [3], optical switches [4], energy harvesting applications [5,6,7] and microrobotics [8], etc.

Compliant mechanisms are widely used in MEMS structures. Clamped-clamped beams are well-known candidates to fulfill bistability thanks to their outstanding mechanical properties. However, a single straight beam should be axially compressed beyond the critical buckling stress so that it can exhibit two discrete stable positions. At submillimeter scale, stress control is hard to realize in monolithic structures. Thus, pre-shaped beams with initial curved shape are a suitable choice when certain structural conditions are met. This will be discussed further in detail below.

For applications such as switching a miniaturized beam between two discrete positions, the total displacement is a key feature of the actuator. Therefore, the output displacement of micromachined bistable actuators is defined by the design, ruling out position control or additional position sensors.

Various methods and tools can be used to design bistable mechanical actuators. Finite element analysis (FEA) is a common tool to predict the performance of complex MEMS structures. However, in this paper, analytical approaches were used to model mechanical properties of bistable pre-shaped beams for their relatively simple design.

Based on the mechanical theory [9], dedicated analytical tools [10,11,12] have been specifically developed for curved beam snap-through (bistable) actuation. In low-frequency buckling analysis, there has been little research on centro-symmetric modes in bistable beams [13,14,15,16,17,18,19,20,21,22,23] because degree-of-freedom cancelling mechanisms (central shuttle, guiding slider, etc.) are systematically integrated in the design. Thus, centro-symmetric modes are supposed to be inhibited during snap-through. However, these mechanisms are only theoretically effective, and are subject to functional failure if the actuation component or the testing device cannot fully contain *z*-axis rotation (due to small contact area, assembly mismatch, probe tip form, insufficient fixture, etc.) when the beams are switching stable positions. This paper assesses the impact of anti-symmetric modes on bistable pre-shaped double beams.

Bistable actuators have been fabricated, either at meso-scale [24,25] or micro-scale, generally by silicon technologies [26,27,28,29,30,31,32,33]. The present work describes the design, fabrication and test of bistable micro-actuators composed of pre-shaped buckled beams in silicon. These units are integrated in an array for collaborative horizontal displacement as a micro-conveyance system. Size reduction of buckled beam actuators is often difficult to obtain. In particular, horizontal switching structures lead to the design of long and slender buckled beams with a relatively high aspect ratio. Deep reactive ion etching (DRIE) is suitable to achieve such structures. Yet the deviation in verticality of sidewalls etched by DRIE can lead to significant difference between design and experimental results [13], since large-stroke slender structures may not allow to design patterns able to guarantee etching area uniformity. The designed structure is supposed to fit one buckling mode composition, namely mode 1 + 3, thanks to the shuttle connecting the center of double parallel beams, which inhibits the presence of mode 2. Therefore, the classic analytical model assuming rectangular beam cross-section should be revised.

However, a centro-symmetric beam form was observed and registered from video recording images of bistable actuator switching test, as well as a sudden fall of actuation force in experimental data during snap-through. These phenomena strongly suggest that another mode composition (mode 1 + 2) arises during actuation with modified buckling form. Since the previous analytical model does not take centro-symmetric mode into account, a new refined model has to be established to predict the restoring force and the deflected beam form.

This paper presents a refined model of a bistable buckled beam actuator with theoretical approaches based on experimental data analysis. Buckled beams were fabricated according to the design and then characterized. The model includes effective geometric features of processed structures and mode superposition. It provides new design rules for buckled beams actuators.

## 2. Design

This section discusses the corresponding models to determine the size of a silicon-based bistable buckled beam actuator. Bistability is achieved by beam buckling. The buckling effect of beams under axial loading has been described in [12]. Because microstructures should be functional right after fabrication, pre-shaped buckled beams are chosen.

**Figure 1:**
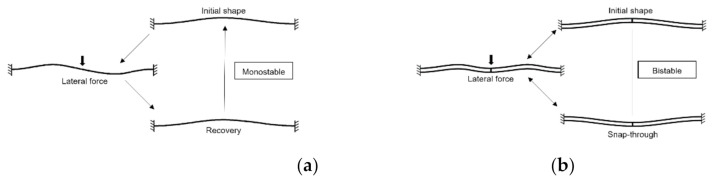
Clamped-clamped pre-shaped beams: (**a**) single pre-shaped beam; (**b**) double pre-shaped buckled beams adapted from [12].

The studied structure is a sine-shaped single beam clamped at both ends. When a force is applied at the center of the beam in the transverse direction, its stiffness makes it turn back to the initial position if the force is removed (Figure 1). Thus, a bistable actuator cannot be obtained with a single beam design.

A structure with double pre-shaped beams, connected at their center and clamped at both ends was proposed in [12]. This double-beam structure forms a bistable actuator. The difference of the buckling modes of single and double beams can be explained in Figure 1.

**Figure 2:**
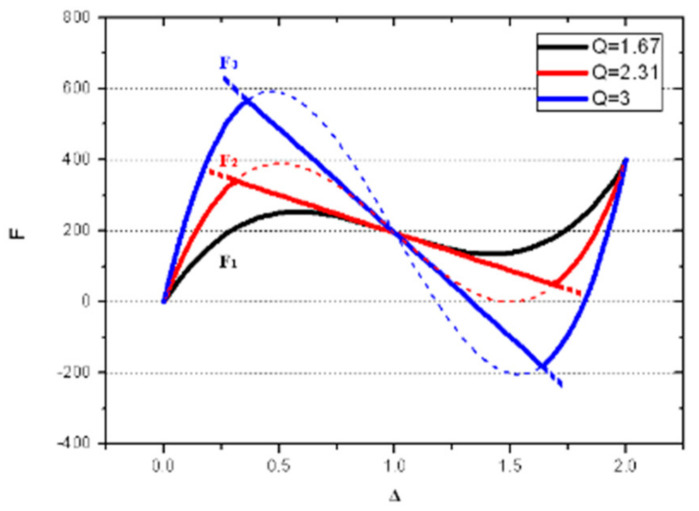
Normalized restoring force as a function of the normalized displacement for mode 1 only (F_1_: black curve), mode 1 + mode 2 (F_2_: red curve) and mode 1 + mode 3 (F_3_: blue curve).

An analytical model was introduced in [12], based on mode superposition. It is commonly considered that the buckling modes of a pre-shaped buckled beam clamped at both ends form an orthogonal set of trigonometric functions. This set is implemented in the superposition theory of buckling modes of pre-shaped beams, with the same boundary conditions. The buckling analysis can be reduced to the first three modes. In the case of pre-shaped beams, the solution is not unique but depends on the axial stress during deflection. Figure 2 represents three normalized force-displacement curves showing the presence of mode 1, mode 2 and mode 3 at different critical values of the apex height over width ratio Q. The curve F_1_ represents the case with small Q values, which only exhibits mode 1. The other two curves, F_2_ and F_3_, both have a centered linear zone where the combination of mode 1 and mode 2 (respectively mode 1 and mode 3) is present, with mode 1 only outside the linear zone.

The buckling mode 2 deserves special attention: its innate centro-symmetric nature tends to bring the deformed beam back to its initial position, as long as the shape amplitude remains at a relevant scale. Theoretically, the shuttle between two parallel pre-shaped beams (see Figure 1) mechanically inhibits the buckling mode 2 without affecting symmetric modes (mode 1, mode 3, etc.).

## 3. Characterization

**Figure 3:**
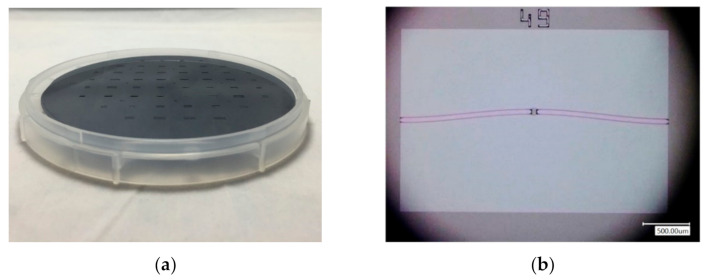
Microfabricated structures: (**a**) SOI wafer with beam structures on the device layer; (**b**) a bistable pre-shaped double beam under an optical microscope.

By considering process specifications, a series of beam structures were proposed. The beam length ranges from 2 to 4 mm, width from 5 to 20 μm and the central apex from 20 to 100 μm. The beams were fabricated on the 50-μm device layer of an SOI wafer, thus featuring a height of 50 μm. A total of 10 double pre-shaped beams of aforementioned sizes received batch fabrication. The predicted maximum restoring lateral force of the beams is 0.02–13.1 mN (Figure 3).

**Figure 4:**
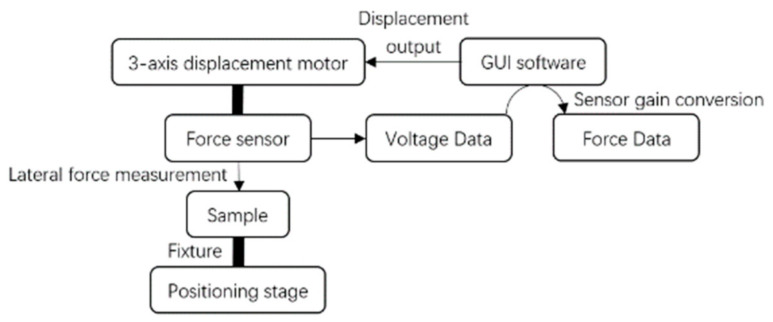
Characterization setup on the Femtotools^®^ MEMS test station.

**Figure 5:**
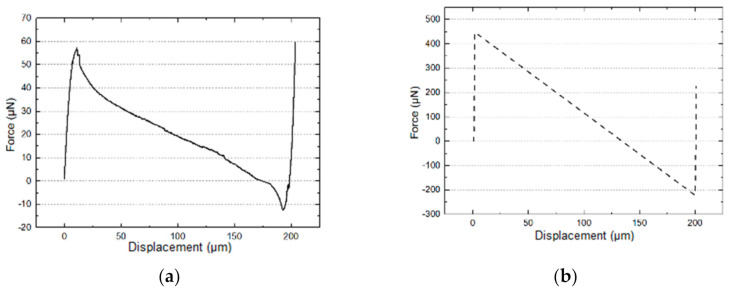
Restoring force as a function of displacement for sample No. 1: (**a**) experimental data; (**b**) analytical result.

A Femtotools^®^ MEMS test station was used for characterization (Figure 4). A lateral force probe (FT-S1000-LA), able to apply forces up to 1000 μN with a resolution of 0.05 μN (at 10 Hz) and an attack angle at 45° in the vertical plane was used. Figure 5 shows the measured force-displacement curve for a sample comprising two 3-mm long, 5-μm wide and 100-μm central apex pre-shaped beams. The measured stroke is 198 μm, and the measured maximum force is 57 μN. The stroke measurement matches both analytical and FEM results (198 μm by characterization vs. 200 μm by analytical calculation and FEM). However, the measured (57 μN) and the analytical (440 μN) maximum force differ by almost one order of magnitude.

The same measurements were made on all 10 samples: similar characteristics were observed. The measured stroke is very close to analytical results, while all the samples exhibit a measured maximum force near 10 times smaller than the analytical value.

Since the analytical model for pre-shaped beams [12] cannot be applied to this case, a modified version taking into account beams with trapezoidal-form cross section should be established.

### 3.1. Refined Analytical Model

Table 1 recapitulates all parameters used for the refined analytical model.

**Figure 6:**
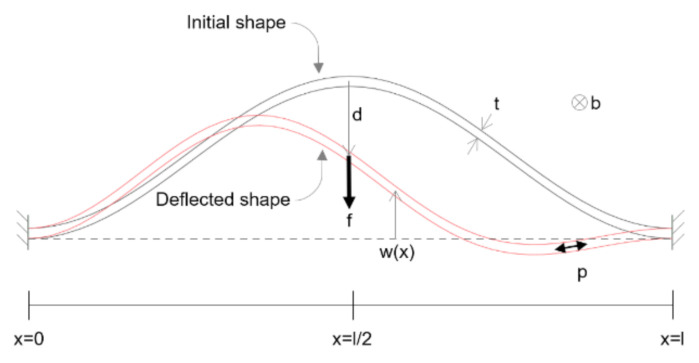
Geometry and notation for the analytical model.

As shown in Figure 6, the deflected shape of pre-shaped beams w(x) can be considered with superposition of buckling modes $w_{j}$:

(1) $$w\left(x\right)\;=\;\overset{\infty }{\underset{j=1}{\sum }}a_{j}w_{j}\left(x\right)$$

As a critical parameter of the buckling behavior of the beams, the constant Q is the geometric ratio between the apex height h and the width t of a pre-shaped beam:

(2) $$Q\;=\;\frac{h}{t}$$

The normalization of certain parameters is defined as follows

(3) $$\Delta \;=\;\frac{d}{h},\;W\left(X\right)\;=\;\frac{w\left(x\right)}{h},\;X\;=\;\frac{x}{l},\;N^{2}\;=\;p\frac{l^{2}}{\mathrm{EI}},\;S\;=\;s\frac{l}{h^{2}},\;F\;=\;f\frac{l^{3}}{\mathrm{EIh}}$$

Specifically

(4) $$W\left(X\right)\;=\;\overset{\infty }{\underset{j\;=\;1}{\sum }}A_{j}W_{j}\left(x\right)$$

**Figure 7:**
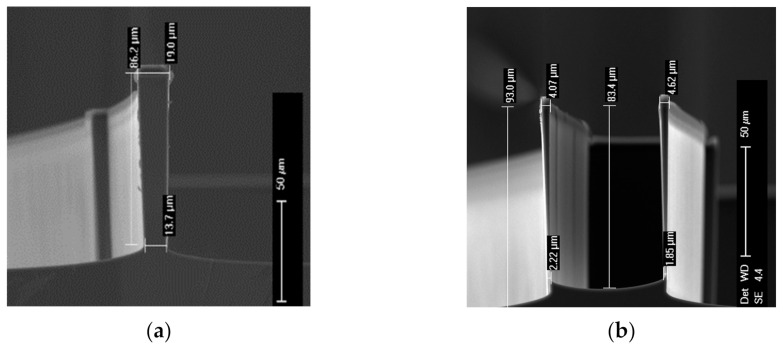

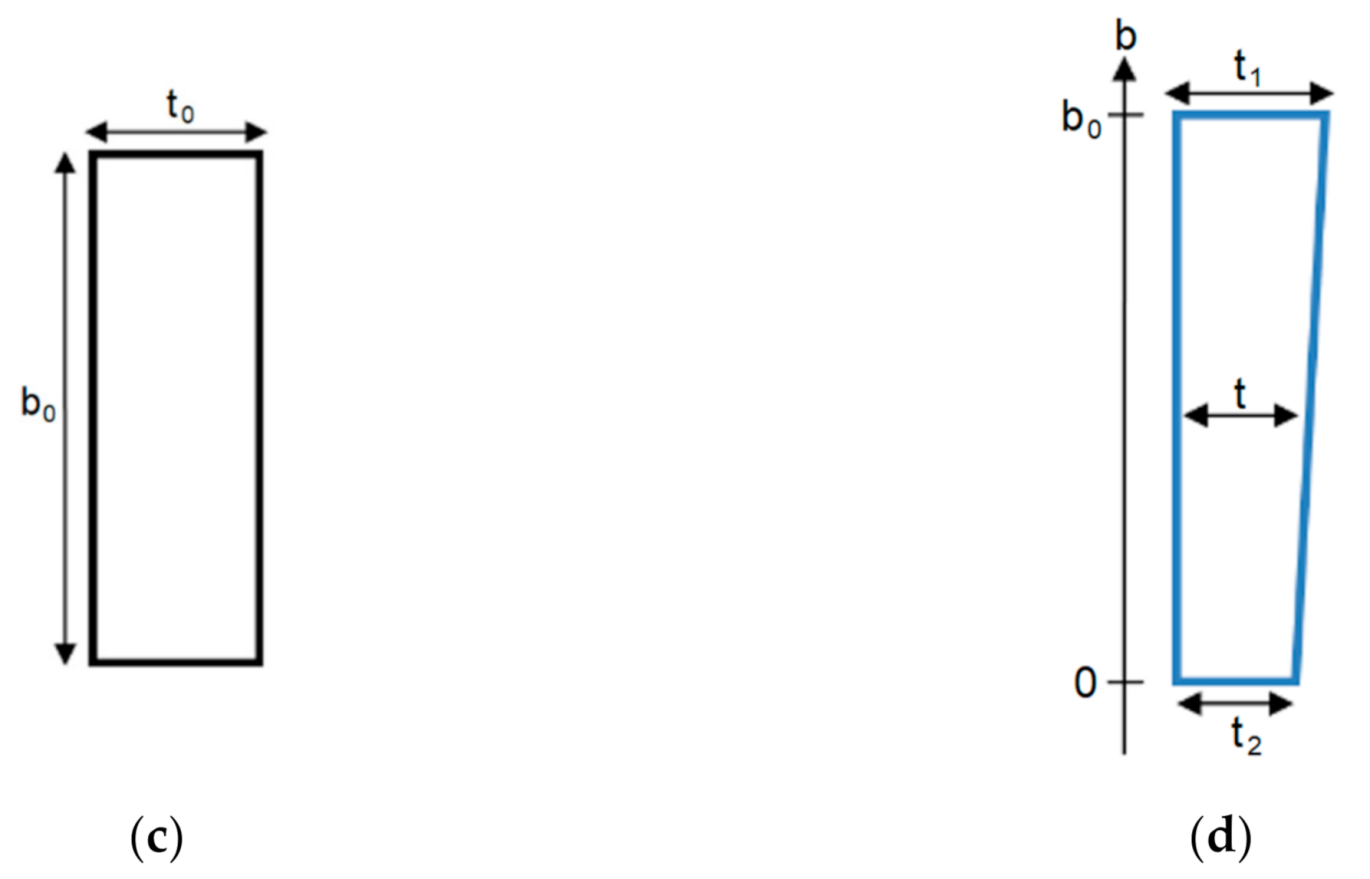
Investigation and modeling of the cross-section shape of real beam structures: (**a**,**b**) SEM views of Si only DRIE etching showing the verticality of beam sidewalls; (**c**) rectangular shaped and (**d**) trapezoidal shaped beam cross-section.

The method for the analytical model of [12] remains relevant to the studied beams. For Q > 6, the maximum restoring force of a double pre-shaped buckled beam actuator with rectangular section (Figure 7c) can be expressed in a simplified form:

(5) $$F_{\max}\;=\;8\pi^{4}\frac{{\mathrm{Ehb}}_{0}t_{0}^{3}}{12l^{3}}$$

Nevertheless, the trapezoidal geometry (Figure 7d) requires re-writing the equations in order to fit real geometric features obtained by the DRIE process.

For the trapezoidal cross-section, the thickness b is a function of the width t in the following linear equation:

(6) $$b\left(t\right)\;=\;b_{0}\frac{t\;-\;t_{2}}{t_{1}\;-\;t_{2}}$$

Consider a trapeze of top width $t_{1}$ and bottom width $t_{2}$ ($t_{1}\;$ > ${\;t}_{2}$). Such beam shape corresponds to a maximum restoring force of the micro-actuator

(7) $$F_{\mathrm{refined}}\;=\;\int_{0}^{t_{0}}8\pi^{4}\frac{{\mathrm{Eht}}^{3}}{12l^{3}}\mathrm{db}\;=\;\int_{w_{2}}^{w_{1}}\frac{2\pi^{4}{\mathrm{Eht}}_{0}}{3l^{3}}\frac{t^{3}}{t_{1}\;-\;t_{2}}\mathrm{dt}$$

Therefore, we obtain

(8) $$F_{\mathrm{refined}}\;=\;\frac{\pi^{4}{\mathrm{Ehb}}_{0}}{6l^{3}}\frac{t_{1}^{4}\;-\;t_{2}^{4}}{t_{1}\;-\;t_{2}}$$

The restoring force can then be expressed as a function of the effective width $t_{\mathrm{eff}}$:

(9) $$F_{\mathrm{refined}}\;=\;8\pi^{4}\frac{{\mathrm{Ehb}}_{0}t_{\mathrm{eff}}^{3}}{12l^{3}}$$

From Equations (8) and (9), the effective width t_eff_ can be deduced as a function of the geometric parameters of the modeled shape:

(10) $$t_{\mathrm{eff}}\;=\;{\left(\frac{1}{4}\left(t_{1}\;+\;t_{2}\right)\left(t_{1}^{2}\;+\;t_{2}^{2}\right)\right)}^{1/3}$$

### 3.2. Application in Fabricated Structures

The modified model was applied to the sample described in Figure 5. Considering the measured beam sidewall angle (88°) by observing the SEM image of a cleaved sample, a width at the bottom of beams $t_{2}$ = 1 μm is estimated. Since all the micro-actuators were processed on the same wafer, with a top width $t_{1}$ = 4 μm for all beams, they should share the same effective width, which is 2.77 μm.

**Figure 8:**
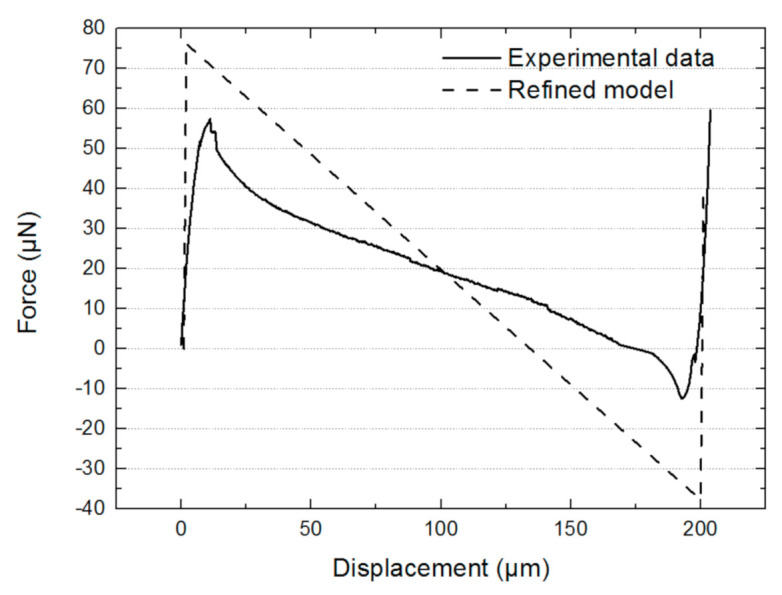
Experimental and refined model results ($t_{2}$ = 1 μm) of the restoring force as a function of displacement.

By using the effective width, the previous experimental curve is then compared to the predicted results from the first refined analytical model. The curve of the new model and the experimental curve are plotted in Figure 8. The maximum restoring force obtained by the modified model is 76 μN. This modified value is much closer to the experimental one.

### 3.3. Anti-Symmetric Mode

**Figure 9:**
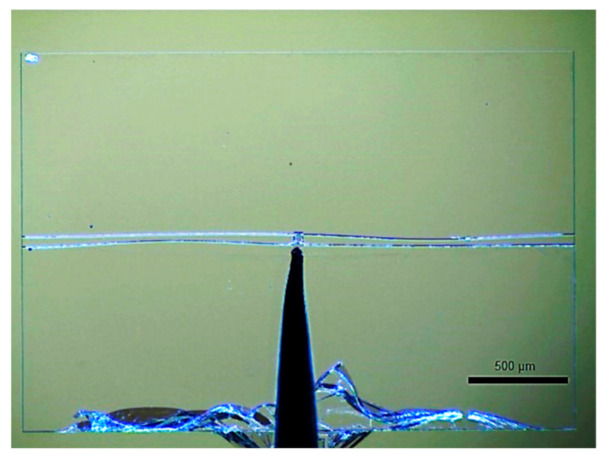
Top view of the anti-symmetric form of the beams when the actuator attained the midpoint of its stroke.

Theoretical models assume that the central shuttle of the double beams is sufficient to inhibit mode 2. However, the experimental results show that an anti-symmetric mode did occur during the measurement (Figure 9). Since the entire wafer had to be fixed on the platform of Femtotools^®^ with 2 simple clips, manual alignment had to be performed under a microscope to visually center the measured sample in front of the force probe. A small but noticeable angular offset was observed at the contact zone, which generated an unexpected torque at the center of the beams. This obviously contradicted the hypothesis assumed above and mentioned in [12]. The following analysis exhibits its situational presence when certain conditions are met.

**Figure 10:**
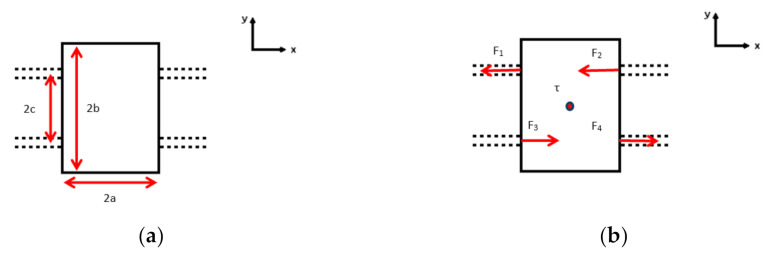
Top view of the central shuttle with 4 beam branches in dashed lines: (**a**) dimension parameters; (**b**) constrained by a torque and lateral forces.

In Figure 10a, the top view of the central shuttle is represented by a 2a × 2b rectangle with four branches 1, 2, 3 and 4 in dashed lines. The double beams are separated by a distance of 2c. Consider a torque applied at the center of the central shuttle, which is noted as τ (Figure 10b). In response to such torque, four reaction forces named respectively as F_1_, F_2_, F_3_ and F_4_ occur at each beam-shuttle interface for the four branches. As the reaction forces are relatively small, they are perpendicular to the side lines of the central shuttle. Thus, static mechanical equilibrium can be achieved by the following equations:

(11) $$-F_{1}\;-\;F_{2}\;+\;F_{3}\;+\;F_{4}\;=\;0,$$

(12) $$𝜏\;-\;F_{1}c\;-\;F_{2}c\;-\;F_{3}c\;-\;F_{4}c\;=\;0$$

The signs of F_1_, F_2_, F_3_ and F_4_ shows the balancing effect of τ.

Since the central shuttle is also centro-symmetric, it yields:

(13) $$F_{1}\;=\;F_{2}\;=\;F_{3}\;=\;F_{4}$$

Hence

(14) $$F_{1}\;=\;F_{2}\;=\;F_{3}\;=\;F_{4}\;=\;\frac{𝜏}{4c}$$

Note that $F_{2}$ and $F_{3}$ are extensive forces that can substantially modify the axial force, which is supposed to be always compressive throughout the stroke. To a greater extent, given the presence of these extensive forces, maximal axial stress is subject to sudden change if a sufficient torque is applied. Based on the aforementioned assumption, we suppose mode 2 appears when there is a torque applied at the center of the structure perpendicular to the horizontal plane, which is responsible for beam distortion. Such torque provokes the rise of mode 2 so that it eventually replaces mode 3. Therefore, the maximal normalized axial stress is no longer $N_{3}^{2}$ but rather $N_{2}^{2}$ [6].

(15) $$\left\{\begin{array}{c} N_{2}^{2}\;=\;8.18\pi^{2}\\ \;N_{3}^{2}\;=\;16\pi^{2} \end{array}\right.$$

However, the observed centro-symmetric mode is not identical to mode 2 in terms of deflected shape, as seen in Figure 9. This phenomenon can be explained by the small variation of axial stress in beams. In fact, as the two beams are separated by a sizeable distance (2c = 50 μm), the central shuttle, rotated by a certain torque and constrained by mechanical reactions, generates additional stress, be it compressive or tensile, in all branches of both beams.

Given the general expression of normalized axial stress introduced in [12]:

(16) $$\frac{N^{2}}{12Q^{2}}\;=\;\frac{N_{1}^{2}}{16}\;-\;\overset{n}{\underset{j\;=\;1}{\sum }}\frac{A_{j}^{2}N_{j}^{2}}{4}$$

Considering that only modes 1 and 2 are preserved by neglecting higher modes, Equation (16) can be rewritten as: As a matter of fact, the equation above can be simplified with the presence of only mode 1 and mode 2:

(17) $$\frac{N_{2}^{2}}{12Q^{2}}\;=\;\frac{N_{1}^{2}}{16}\;-\;\frac{A_{1}^{2}N_{1}^{2}}{4}\;-\;\frac{A_{2}^{2}N_{2}^{2}}{4}$$

Since $A_{1}$ only depends on Δ [12]:

(18) $$A_{1}\;=\;\frac{1\;-\;\Delta }{2}$$

Therefore

(19) $$A_{2}^{2}\;=\;-\frac{1}{3Q^{2}}\;+\;\frac{N_{1}^{2}}{4N_{2}^{2}}\left(2\Delta \;-\;\Delta^{2}\right)$$

The values of Δ can only be taken with $A_{2}^{2}$ > 0. In particular, $A_{2}^{2}$ takes maximum value when Δ = 1, which is the semi-stroke of the structure.

(20) $$A_{2\max}^{2}=\;-\;\frac{1}{3Q^{2}}\;+\;\frac{N_{1}^{2}}{4N_{2}^{2}}$$

On the other side, the deflected shape of the beams can be described by the following equation introduced in [12]:

(21) $$w\left(x\right)\;=\;a_{1}w_{1}\left(x\right)\;+\;a_{2}w_{2}\left(x\right)$$

As explained previously, the angular deviation of the central shuttle, noted as α, caused extra stress in the parts of both beams. Its impact should be assessed by deriving w(x) at its midpoint:

(22) $$\frac{d}{\mathrm{dx}}w\left(l/2\right)\;=\;a_{1}\frac{d}{\mathrm{dx}}w_{1}\left(l/2\right)\;+\;a_{2}\frac{d}{\mathrm{dx}}w_{2}\left(l/2\right)$$

As

(23) $$\left\{\begin{array}{c} \frac{d}{\mathrm{dx}}w_{1}\left(x\right)\;=\;\frac{a_{1}N_{1}}{l}\sin\left(N_{1}\frac{x}{l}\right)\\ \frac{d}{\mathrm{dx}}w_{2}\left(x\right)\;=\;a_{2}\left(-\frac{2}{l}\;+\;\frac{N_{2}}{l}\sin\left(N_{2}\frac{x}{l}\right)\;+\;\frac{2}{l}\cos\left(N_{2}\frac{x}{l}\right)\right) \end{array}\right.$$

We define the theoretical angle β

(24) $$\beta ≅\tan\beta \;=\;\left|\frac{a_{2}}{l}\left(-2\;+\;N_{2}\sin\left(\frac{N_{2}}{2}\right)\;+\;2\cos\left(\frac{N_{2}}{2}\right)\right)\right|$$

β is then compared to α for the following three cases:

1. If α < β, branch 1 and branch 4 have extra compressive stress while branch 2 and branch 3 have extra tensile stress;
2. If α = β, no extra stress is applied;
3. If α > β, branch 1 and branch 4 have extra tensile stress while branch 2 and branch 3 have extra compressive stress.

**Figure 11:**
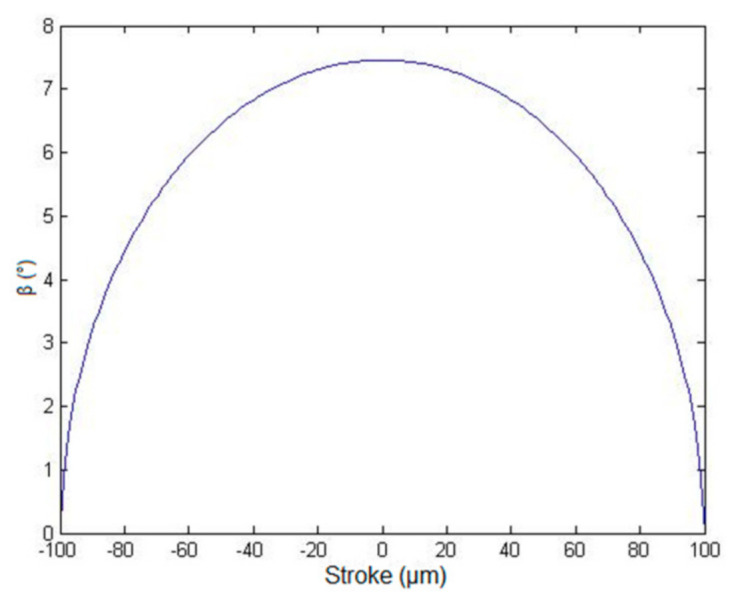
Theoretical angle β as a function of the stroke.

In Figure 11, as β is proportional to $a_{2}$, the maximum value of β is achieved when the structure is displaced at exactly its semi-stroke: $\beta_{\max}\approx 7{}^{\circ}$.

Since 7° is very small, consider case 3, where the extra stress $\sigma_{\mathrm{ex}}$ and its normalized form $N_{\mathrm{ex}}^{2}$ can be expressed:

(25) $$\sigma_{\mathrm{ex}}\;=\;E\frac{\left(\alpha \;-\;\beta \right)c}{2l}$$

(26) $$N_{\mathrm{ex}}^{2}\;=\;\delta \sigma \;\cdot \;\mathrm{bt}\frac{l^{2}}{\mathrm{EI}}\;=\;6\left(\alpha \;-\;\beta \right)\frac{\mathrm{cl}}{t^{2}}$$

It is worth noting that the sign of $N_{\mathrm{ex}}^{2}$ depends on the type of generated extra stress. It is positive if the stress is tensile and can be negative if the stress is compressive.

Consider the total normalized axial stress by adding $N_{\mathrm{ex}}^{2}$ and $N_{2}^{2}$:

(27) $$N^{2}\;=\;N_{\mathrm{ex}}^{2}\;+\;N_{2}^{2}$$

Equation (16) can be expressed with modified $A_{2}$ in branch k (k = 1, 2, 3 and 4), noted as ${A^{\prime }}_{2,k}^{2}$:

(28) $$\frac{N_{\mathrm{ex}}^{2}\;+\;N_{2}^{2}}{12Q^{2}}\;=\;\frac{N_{1}^{2}}{16}\;-\;\frac{A_{1}^{2}N_{1}^{2}}{4}\;-\;\frac{{A^{\prime }}_{2,k}^{2}N_{2}^{2}}{4}$$

The difference between Equations (17) and (28) gives:

(29) $$\frac{{N_{k}^{2}}_{\mathrm{ex}}}{12Q^{2}}\;=\;\frac{A_{2,k}^{2}N_{2}^{2}}{4}\;-\;\frac{{A^{\prime }}_{2,k}^{2}N_{2}^{2}}{4}$$

Thus

(30) $${A^{\prime }}_{2,k}^{2}\;=\;A_{2,k}^{2}\;-\;\frac{{N_{k}^{2}}_{\mathrm{ex}}}{3N_{2}^{2}Q^{2}}$$

## 4. Discussion

Given Equation (30), we find that:

1. if ${N_{k}^{2}}_{\mathrm{ex}}$ is negative, the corresponding branch is more curved;
2. if ${N_{k}^{2}}_{\mathrm{ex}}$ is positive, the corresponding branch is less curved, or can even be flattened when ${A^{\prime }}_{2,k}^{2}$ = 0.

**Figure 12:**
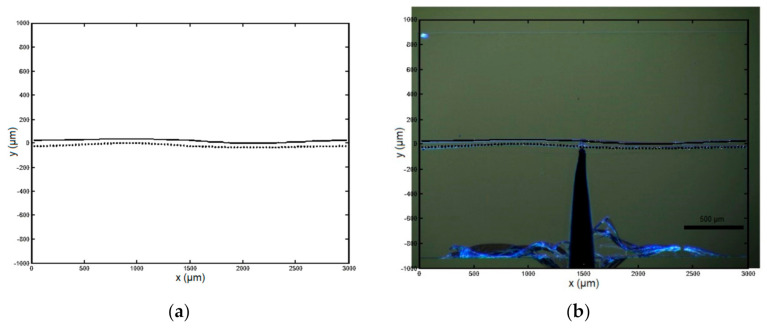
Numerical result of the buckling mode for the measured actuator at the midpoint of its stroke: (**a**) standalone modeling result; (**b**) superposition with experimental image shown in Figure 9.

In Figure 12, the two beams are closer at the two anti-nodes because of amplitude modification of mode 2. A contact angle of α = 12°, has been included in the calculation. The numerical result agrees with image taken from experiments.

**Figure 13:**
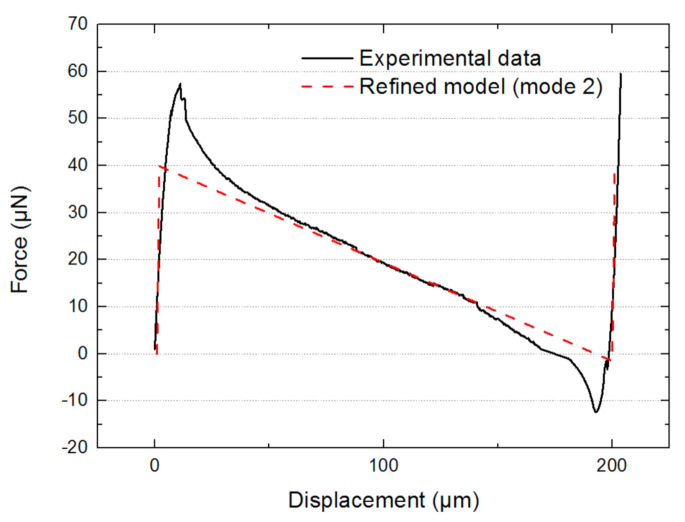
Restoring force as a function of displacement: experimental data and results of the refined model with mode 2.

Next, the experimental data have been compared to the model using a combination of mode 1 and mode 2 (Figure 13).

The maximum force determined by the new refined model is smaller than the experimental one (57 μN by experimental data vs. 40 μN by analytical results). The central part of the analytical model almost perfectly fits with the experimental. This result indicates that mode 2 appeared during the switching displacement. In addition, the measured maximum force (57 μN) is very close to the mean value of the maximum force of the refined model with mode 1 + 3 (76 μN) and the one with mode 1 + 2 (40 μN), which is 58 μN. This coincidence can be explained by a possible coexisting 1 + 2 and 1 + 3 combination at the beginning and the end of snap-through. This is achieved by applying a small torque on the shuttle so that the resultant axial force in the two compressive branches is $N_{3}^{2}$ while the resultant axial force in the other two extensive branches is $N_{2}^{2}$. The nonlinear effect shows the mode 1 + 3 quickly vanishes due to the presence of increasing central rotation in the branches with mode 1 + 2, and therefore only mode 1 + 2 is preserved at the center of the curve.

## 5. Conclusions

A series of bistable micro-actuators based on pre-shaped buckled beams have been designed, fabricated and characterized. The analytical results using models from the literature lead to significant difference between theoretical lateral restoring force and experimental results. A refined analytical model, including the real fabricated trapezoidal shape of the beams, have been developed, and results of the model have been found to be in relatively good agreement with microfabricated features.

The remaining difference between modeled and experimental results has been attributed to a combination of buckling modes 1 and 2. With significant difference of axial forces in the beams, mode 2 and mode 3 coexist at the beginning and the end of the snap-through. However, mode 3 diminishes due to increasing rotation of the central shuttle. Thus, only mode 1 + 2 is present at the mid-stroke. Combining real microfabricated shapes and mode superposition, a very good agreement was obtained between modeling and experiment.

The results obtained confirm for the first time in the literature that modeling can closely predict the experimental switching behavior of real microfabricated curved beams.

## Figures and Tables

**Table 1:** Summary of the parameters of the refined model.

Notation	Parameter
b	Thickness of the beam
t	Width of the beam
l	Length of the beam
h	Apex height of the pre-shaped beam
Q	Geometric factor of the beam
d	Central deflection of the beam
w	Deflected shape of the beam
x	Longitudinal coordinates
E	Young’s modulus
I	Moment of inertia of the beam
p	Axial force in the beam
s	Total length of the beam
f	Actuation force
Δ	Normalized central deflection
W	Normalized deflected shape
X	Normalized longitudinal coordinates
N^2^	Normalized axial stress
S	Normalized total length
F	Normalized actuation force
